# Oral health–related quality of life of patients with oral chronic graft-versus-host disease

**DOI:** 10.1007/s00520-021-06197-7

**Published:** 2021-04-21

**Authors:** Juliette Stolze, Marlou Boor, Mette D. Hazenberg, Henk S. Brand, Judith E. Raber-Durlacher, Alexa M. G. A. Laheij

**Affiliations:** 1 Department of Oral Medicine, Academic Centre for Dentistry (ACTA), University of Amsterdam and Vrije Universiteit Amsterdam, Gustav Mahlerlaan 3004, 1081 LA Amsterdam, The Netherlands; 2 Department of Oral Biochemistry, Academic Centre for Dentistry (ACTA), University of Amsterdam and Vrije Universiteit Amsterdam, Amsterdam, The Netherlands; 3 Department of Hematology Amsterdam UMC, University of Amsterdam, Amsterdam, The Netherlands; 4 Department of Oral Maxillofacial Surgery, Amsterdam UMC, University of Amsterdam, Amsterdam, The Netherlands; 5 Department of Preventive Dentistry, Academic Centre for Dentistry (ACTA), University of Amsterdam and Vrije Universiteit Amsterdam, Amsterdam, The Netherlands

**Keywords:** Oral chronic-graft-versus-host disease, GVHD, OHRQoL, OHIP-14, Quality of life, Hyposalivation, Xerostomia

## Abstract

**Purpose:**

Symptoms of oral chronic graft-versus-host-disease (cGVHD) may significantly affect the oral health–related quality of life (OHRQoL). This study aimed to assess the OHRQoL in patients with oral cGVHD and to examine whether oral cGVHD symptoms, mucosal cGVHD, and salivary gland function correlated with OHRQoL.

**Methods:**

Patients referred to the oral cGVHD outpatient clinic were included. Severity of oral mucosal cGVHD, oral cGVHD symptoms, and OHRQoL was assessed by the NIH OMS, NIH OSS, and OHIP-14, respectively. Unstimulated and stimulated whole salivary flow rates were determined and categorized into “hyposalivation,” “normal salivary flow,” and “hypersalivation.”

**Results:**

Of 56 included patients, 80% had mild, moderate, or severe oral mucosal cGVHD. Mean total score of OHRQoL was 16.5 (±11.7), negatively affected by functional problems. Patients reported highest scores regarding oral sensitivity and xerostomia. Significant correlations were found between severity of oral pain and OHRQoL and between oral sensitivity and OHRQoL. No correlation was found between oral mucosal cGVHD and OHRQoL. Patients with hyposalivation, normal salivary flow, and hypersalivation reported equal levels of OHRQoL.

**Conclusion:**

Results demonstrate that the OHRQoL was mostly negatively affected by complaints of oral pain and oral sensitivity and less by the severity of oral mucosal cGVHD assessed by the NIH OMS score. Special attention of (oral) health care professionals for patients with oral cGVHD is mandatory to alleviate their symptoms and improve OHRQoL.

## Introduction

Hematopoietic stem cell transplantation (HSCT) is a widely used intervention to treat malignancies and other disorders of the hematopoietic system. The numbers of HSCTs performed continue to rise with more than 45,000 transplants reported in Europe in 2017, of which 18,281 were allogeneic HSCT (with donor-derived cells) [1]. With the introduction of less toxic reduced intensity and non-myeloablative preparative regimens for HSCT [2], HSCT also became a treatment option for older patients and patients with comorbidities.

While the survival rate of allogeneic HSCT is improving, the prevalence of graft-versus-host disease (GVHD) increases [3]. GVHD is a common and complex complication of allogeneic HSCT [4]. In patients with GVHD, a series of alloimmune reactions occur due to a mismatch in HLA antigens [5, 6] leading to acute or chronic GVHD. Nowadays, the distinction between acute and chronic GVHD (cGVHD) is made based on clinical manifestations, rather than based on time after HSCT with a classic dividing line at 100 days [7, 8]. Whereas acute GVHD typically presents specific clinical and histopathological features of the skin, liver, and/or gastrointestinal mucosa, chronic GVHD is defined as a systemic disease with multiorgan involvement, affecting the skin, mouth, genitals, eyes, liver, or lungs, presenting a wide range of signs and symptoms [5, 9–11]. Chronic GVHD is associated with increased morbidity and mortality. The 5-year non-relapse mortality rate of cGVHD ranges from 5 to 70% depending on factors such as prior acute GVHD, time between transplantation and development of cGVHD, donor type, and gender mismatch [12].

The incidence of cGVHD ranges between 30 and 70% depending on differences in age, type of donor, and post-HSCT immunosuppressive therapy [5, 11, 13, 14]. Clinical features of cGVHD mimic multiple autoimmune or immune-mediated conditions characterized by chronic inflammation [5]. The oral cavity is one of the most frequently affected anatomical sites [7]. Up to 80% of patients with cGVHD demonstrate oral involvement at a certain time point after HSCT [15, 16]. Oral manifestations can be divided into mucosal disease, and/or salivary gland dysfunction and/or, less frequently, sclerotic changes [17]. Signs and symptoms include sensitivity of the oral mucosa to foods and liquids, oral pain, xerostomia and/or hyposalivation, increased risk for dental caries, difficulty speaking, recurrent fungal infections, recurrent superficial mucoceles, and mucosal presentations such as erythema, lichenoid or ulceration, taste alterations, dysphagia, and reduced mouth opening [7, 16]. These complications may significantly affect the Oral Health–related Quality of Life (OHRQoL) of GVHD patients [18–20].

Due to the increased number of HSCT’s performed and the increased survival rate after treatment, the quality of life (QoL) of patients has become an important health outcome. However, to what extent oral cGVHD affects the OHRQoL of patients is still unclear [10, 17]. Therefore, the aim of this study was to examine the OHRQoL of patients with oral cGVHD and to correlate it to oral mucosal cGVHD and salivary gland cGVHD.

## Patients and methods

Recipients of allogeneic HSCT with oral cGVHD complaints referred to the department of Oral and Maxillofacial Surgery, Amsterdam UMC, location AMC, between September 2015 and December 2019 were included. Data concerning gender, age, time since HSCT, type of donor, and preparative conditioning regimen were retrieved from the medical records. During a clinical exam, the severity of oral mucosal cGVHD was assessed by a specialized dentist (JR-D), and patients were asked to fill out questionnaires about oral cGVHD symptoms and OHRQoL. Unstimulated and stimulated whole salivary flow rates were measured. The Ethics Review Committee of the Amsterdam UMC, location AMC, confirmed that the Medical Research Involving Human Subjects Act (WMO) does not apply to this study (W15_294 no. 15.0349). All patients provided written informed consent.

### Oral cGVHD assessment

For assessment of the severity of oral mucosal cGVHD, the NIH cGVHD Oral Mucosal Score (NIH OMS) was used [19, 20]. The NIH OMS assesses the extent and severity of erythema, lichenoid lesions, ulcerations, and mucoceles in the oral cavity. The total score, ranging from 0 “no mucosal changes” to 15 “severe oral mucosal changes,” is divided into four categories “no GVHD” (score 0), “mild” (scores 1–3), “moderate” (scores 4–9), and “severe” (scores 10–15).

For the assessment of patient-reported severity of oral cGVHD, patients were asked to complete the NIH Oral Symptom Scores (NIH OSS) [21, 22]. This questionnaire scores xerostomia, oral pain, and oral sensitivity during the last week, each rated on an 11-point scale ranging from 0 “not existing” to 10 “worst imaginable.”

### Salivary flow rates

To determine the presence and severity of salivary gland cGVHD, the unstimulated (UWS) and stimulated whole salivary flow rate (SWS) were measured. For the collection of unstimulated saliva, patients were asked to firstly swallow and thereafter salivate all accumulated saliva into a cup every 30 s during 5 min. For the collection of stimulated saliva, participants were asked to follow the same procedure while chewing on flavorless paraffin gum. Salivary flow rates were determined gravimetrically and expressed as mL/min [23]. The UWS and SWS were categorized into “hyposalivation” (<0.1 mL/min resp. <0.5 mL/min), “normal salivary flow” (0.1–0.5 mL/min resp. 0.5–2.0 mL/min), and “hypersalivation” (>0.5mL/min resp. >2.0 mL/min) [24].

### OHRQoL assessment

To assess the OHRQoL, patients were asked to complete the Dutch version of the Oral Health Impact Profile-14 (OHIP-14). The OHIP-14 comprises 14 items that measure seven domains of impact on patients OHRQoL: functional limitation, physical pain, psychological discomfort, physical disability, psychological disability, social disability, and social handicap [25, 26]. For each item of the OHIP-14, a 5-point Likert scale is used ranging from 0 “never” to 4 “very often” according to the frequency of the impact. The total OHIP-14 score ranges from 0 (excellent OHRQoL) to 56 (worst OHRQoL).

### Statistical analysis

All variables were summarized using descriptive statistics. IBM SPSS version 26 (IBM Inc., Armonk, USA) was used to perform data analyses. The Pearson’s correlation coefficient (*r*) was used to calculate correlations between oral mucosal cGVHD (NIH OMS total score) and oral cGVHD symptoms (NIH OSS scores), between oral cGVHD symptoms and OHRQoL (OHIP-14 total score) and between oral mucosal cGVHD and OHRQoL. The Kruskal-Wallis test was used to analyze differences in OHRQoL between patients with hyposalivation, normal salivary flow, and hypersalivation as determined by UWS and SWS. A *p* value <0.05 was considered statistically significant.

In some cases, it was not possible to conduct some of the clinical assessments for practical reasons. These included time restriction because patients had multiple other examinations scheduled elsewhere in the hospital, patients had eaten just before the planned salivary flow assessment, or lack of a suitable room.

## Results

### Patient characteristics

Fifty-six patients were included. Table 1 shows the characteristics of these patients. There was an almost equal distribution in matched unrelated (MUD) and matched related donor (MRD) transplants. A majority of the patients received a non-myeloablative preparative regimen. For 75% of the patients, the assessments took place between 0 and 4.5 years after HSCT. There was one outlier of >20 years after HSCT.

**Table 1:** Characteristics of patients with oral chronic graft-versus-host disease (cGVHD) at the time of enrollment

Patient characteristics	*n* (%)	Mean ±SD (range)
Age (years)		55.4 ±13.6 (21–72)
Time between HSCT treatment and assessment (months)		44.8 ±54.4 (2–294)
Male	31 (55.4%)	
Female	25 (44.6%)	
MUD	30 (53.6%)	
MRD	26 (46.4%)	
Myeloablative conditioning	10 (17.9%)	
Reduced-intensity/non-myeloablative conditioning	46 (82.1%)	

*HSCT* hematopoietic stem cell transplantation, *MUD* matched-unrelated donor, *MRD* matched-related donor

### Oral cGVHD assessment

**Fig. 1:**
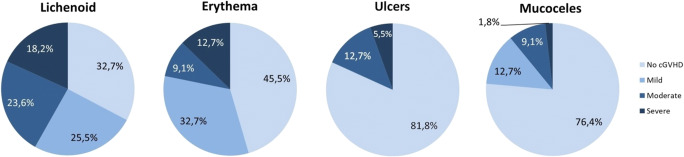
Frequency and severity of lichenoid lesions, erythema, ulcers, and mucoceles in patients with oral chronic graft-versus-host disease (cGVHD) as assessed by NIH OMS (*n*=55)

The total oral mucosal cGVHD score ranged between 0 and 11, with a mean score of 3.2 (±3.0). Most patients had mild (45.5%) or moderate (30.9%) oral mucosal cGVHD. Twenty percent had no oral mucosal cGVHD. Only 2 patients (3.6%) suffered from severe oral mucosal cGVHD. Most patients had lichenoid lesions, of which 25.5% were mild, 23.6% were moderate, and 18.2% severe (Fig. 1). About half of the patients had erythema, of which 32.7% were mild, 9.1% moderate, and 12.7% severe. Of all patients, 12.7% had ulcers involving <20% of the mucosa, and 5.5% had ulcers involving >20% of the mucosa. Mucoceles were reported by a quarter of the patients (24.6%).

**Fig. 2:**
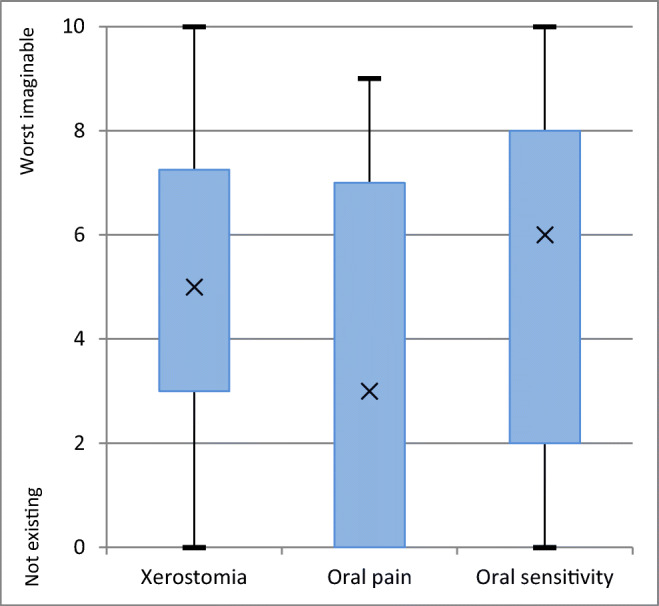
Patient-reported severeness of oral chronic graft-versus-host disease (cGVHD) symptoms as assessed by NIH OSS. Distribution of scores (*n*=44)

Subjective scores for oral cGVHD were available from 44 patients (78.6%) (Fig. 2). Patients reported more severe oral sensitivity (mean 5.2 ±3.0) and xerostomia (mean 5.1 ±3.3) than oral pain (mean 3.4 ±3.2).

### Salivary flow rates

**Fig. 3:**
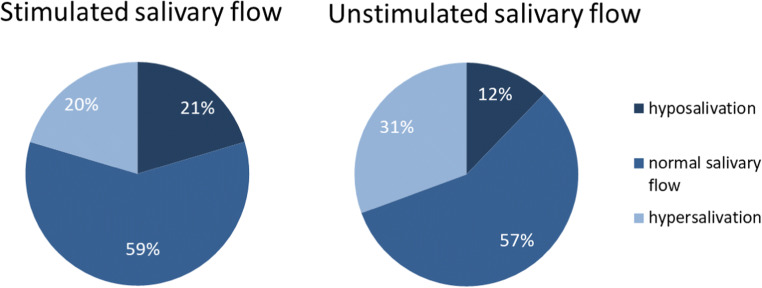
Distribution of the salivary flow rate of oral chronic graft-versus-host disease (cGVHD) patients (*n*=49)

The whole salivary flow rates were assessed for 49 patients (87.5%). The UWS ranged from 0 to 1.6 mL/min, and the SWS ranged from 0 to 5.6 mL/min. Based on UWS and SWS, 12% and 21% of the patients were categorized as hyposalivation (Fig. 3).

### Oral health–related quality of life

**Fig. 4:**
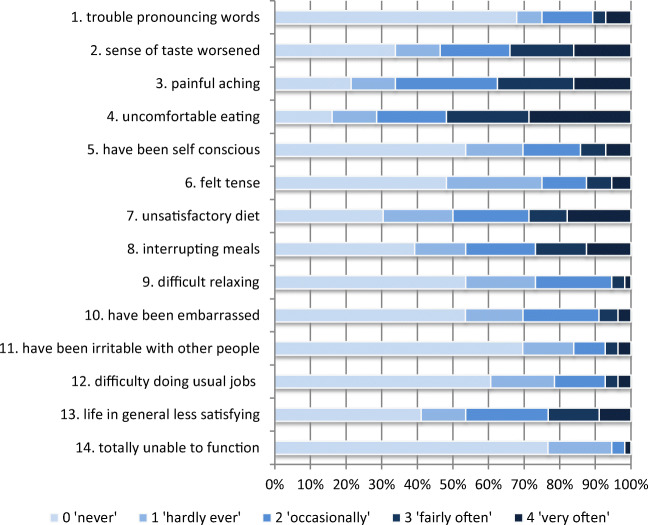
Distribution of the scores on the 14 items of the OHIP-14 of oral chronic graft-versus-host disease (cGVHD) patients (*n* = 56)

The total OHIP-14 scores ranged from 0 to 42 (*n*=56), with a mean score of 16.5 (±11.7). Figure 4 shows the distribution of scores on each of the OHIP-14 items. Items “have you had painful aching in your mouth” and “Have you found it uncomfortable to eat any foods because of problems with your teeth, mouth or dentures” and “Has your diet been unsatisfactory because of problems with your teeth, mouth or dentures?” and “Have you felt that your sense of taste has worsened because of problems with your teeth, mouth or dentures?” received higher scores. Items “Have you been totally unable to function because of problems with your teeth, mouth or dentures?” and “Have you been a bit irritable with other people because of problems with your teeth, mouth or dentures?” gave the least problems for the patients.

### Oral mucosal cGVHD, oral cGVHD symptoms, and OHRQoL

Significant moderate correlations were found between patient-reported severity of oral pain and the OHRQoL (*r*=0.519, *p*<0.0005) and between patient-reported severity of oral sensitivity and the OHRQoL (*r*=0.616, *p*<0.0005). Patients that reported more severe pain and sensitivity of the oral mucosa had a worse OHRQoL. No correlations were found between the severity of oral mucosal cGVHD and the OHRQoL (*r*=0.248, *p*=0.068) and between patient-reported severity of xerostomia and the OHRQoL (*r*=0.026, *p*=0.868).

Significant weak correlations were found between the severity of oral mucosal cGVHD and patient-reported severeness of xerostomia (*r*=–0.312, *p*=0.039), oral pain (*r*=0.313, *p*=0.038), and oral sensitivity (*r*=0.477, *p*=0.001). Patients that had more severe oral mucosal cGVHD may report less severe xerostomia and more severe pain and sensitivity of the oral mucosa.

### Salivary flow rates and OHRQoL

No differences in OHRQoL were found between patients with hyposalivation, normal salivary flow, and hypersalivation as measured by UWS (*p*=0.700) and SWS (*p*=0.104).

## Discussion

This study is one of the first studies that assessed OHRQoL of patients with oral cGVHD using the OHIP-14 questionnaire. Our results demonstrate that the OHRQoL was mostly negatively affected by complaints of oral pain and oral sensitivity and less by the severity of oral mucosal cGVHD. The OHRQoL was affected in particular by functional problems with oral mucosal tissue, the dentition or dentures, like oral pain, uncomfortable eating, or altered taste. Social aspects had less impact on the OHRQoL. A similar pattern was found in a recent systematic review of the OHRQoL in patients with hematological cancers [27]. Functional complications impaired OHRQoL more severely than social aspects.

The OHIP-14 is an internationally validated and widely used questionnaire to assess the OHRQoL. The questionnaire is user friendly and is available in different languages. As a result, comparison is possible with other studies in the literature. To the best of our knowledge, there is no validated questionnaire available specifically designed for evaluating the OHRQoL of patients with oral cGVHD. In our experience, most problems faced by these patients are covered by items from the OHIP-14. The OHIP questions provide a global overview of a person’s perception of the social impact of their oral disorder(s) on their well-being, while at the same time different aspects of impact are addressed. However, designing a specific validated questionnaire for evaluating OHRQoL in patients with oral cGVHD could be considered.

The mean OHIP-14 total score of 16.5 in the present study is relatively high compared to OHIP-14 scores of other hemato-oncologic patients, previously reported, implicating a worse OHRQoL in patients suffering from cGVHD. Mean OHIP-14 total scores of 4.48 [28] and 4.62 [29] have been described for patients with onco-hematologic diseases at admission of or scheduled for HSCT. A mean OHIP-14 score of 10.6 was reported for patients undergoing intravenous chemotherapy [30]. The lower OHIP-14 scores in the previous studies could be explained by the fact that these patients did not suffer from cGVHD or may have received adequate medications (e.g., opioids) to relieve oral pain caused by oral mucositis [31]. These strong analgesics are usually not prescribed to patients with oral cGVHD. On the other hand, it is difficult to interpret or compare the average OHIP total score as no scale has been drawn up in terms of low, moderate, or high OHRQoL. High scores on just two individual domains or items of the OHIP-14 can already indicate a significant effect on the QoL. Therefore, the total score does not do justice to problems that patients may experience in a specific part.

Usually, studies used questionnaires other than the OHIP-14 to assess the (OHR)QoL of cGVHD patients. Several studies reported a reduced QoL of cGVHD patients [10, 32, 33], and correlations between QoL and symptoms of pain and fatigue [18] or xerostomia [16] have been reported as well. In one study among HSCT recipients, patients with extensive cGVHD had on average worse QoL, compared to patients with limited or without cGVHD [34]. In another study among HSCT recipients, of which 33.3% suffered from cGVHD, a reduced QoL was found [35]. Another study reported that the QoL correlated with oral mucositis due to HSCT [36]. However, information about presence or absence of GVHD was not reported in that study. In another study among HSCT recipients of which 69.3% suffered from cGVHD and 35.1% had oral cGVHD, no association between reduced QoL scores and dental disease and symptoms was observed [37].

The OHRQoL was not highly affected for some situations, such as difficulty relaxing or the inability to perform usual jobs. A possible explanation could be the so-called response shift [9]. By having survived cancer and/or because of adaptation and acceptance to a chronic condition, like cGVHD, patients may adapt to their limitations and ultimately perceive better QoL than experienced before [37]. And, in patients with a severe systemic disease, oral health may become less of a priority, and oral symptoms may have less of an impact on the total health-related quality of life (HRQoL) and daily life.

According to Fall-Dickson et al. [17], no reliable clinical signs or related symptoms are known to predict the clinical course of oral involvement of cGVHD. Therefore, they recommended evaluating the impact of oral cGVHD on patients experience/symptoms and (oral health related) QoL. In the present study, oral mucosal cGVHD, assessed by a clinician using the NIH OMS, was significantly correlated with the severity of oral pain and oral sensitivity. However, this correlation was too weak to suggest that oral pain and oral sensitivity may predict the severity of oral mucosal cGVHD. A stronger correlation was found between oral pain and oral sensitivity and the OHRQoL. When determining or predicting the OHRQoL, focus should lie on patient-reported outcomes or symptoms. Since no correlation was found between severity of oral mucosal cGVHD and OHRQoL, clinical manifestations, such as lichenoid and erythema of the oral mucosa, do not necessarily imply worse OHRQoL. A similar result was found in the study of Imanguli et al. [38].

Different studies emphasize the prevalence or severity of xerostomia in HSCT recipients with oral cGVHD. Percentages of patients with xerostomia vary between 43% [16] and 77% [38]. In HSCT patients compared to healthy controls, Brand et al. [39] found a significantly greater severity of xerostomia as well as several other oral complaints. Eighty-six percent of the patients reported having active GVHD or a history of GVHD. However, between patients with or without GVHD, no difference in severity of xerostomia was found. In the present study, patients reported more severe xerostomia than oral pain. A similar result was found by Fall-Dickson and co-workers [16], where xerostomia was more prevalent and severe than oral pain among oral cGVHD patients. The severity of xerostomia was associated with impaired HRQoL, which was also confirmed in another study [16, 38]. Interestingly, in the present study, no correlation was found between the severity of xerostomia and OHRQoL. In addition, the weak negative correlation that was found between the severity of xerostomia and oral mucosal cGVHD may suggest that patients experienced less xerostomia when having more severe oral mucosal cGVHD. However, the clinical relevance may be questioned given the weakness of the correlation. Furthermore, patients with hyposalivation, normal salivary flow, and hypersalivation did not differ in OHRQoL. In contrast, in the study of Imanguli et al. [39], among patients with cGVHD, a significant association was found between salivary gland dysfunction and lower OHRQoL. The low incidence of hyposalivation in the present study (only 12% and 21% based on UWS and SWS, respectively) could explain why no differences could be identified. Findings in the present study confirm the assumption that oral mucosal cGVHD and salivary cGVHD are two distinct manifestations of the disease [15, 40].

Due to the cross-sectional design of the study, all assessments were taken at a single time point, with varying time since HSCT and varying duration of cGVHD. Although all patients were suffering from oral cGVHD, its manifestations are known to be fluctuating in severity over time [4]. Therefore, future studies investigating the impact of oral cGVHD on (OHR)QoL should preferably have a prospective design and follow patients over a longer period of time and should include larger numbers of patients taking potential confounders into account.

To conclude, the results from the present study highlight the important observation that HSCT recipients who suffer from oral cGVHD have a lower OHRQoL. Special, long-term, attention aiming to reduce oral mucosal pain and sensitivity could relieve symptoms and may improve OHRQoL [41, 42].

## Data Availability

The data that support the findings of this study are available on request from the corresponding author.

## Code availability

Does not apply.
